# Relationship between Green Leaders’ Emotional Intelligence and Employees’ Green Behavior: A PLS-SEM Approach

**DOI:** 10.3390/bs13010025

**Published:** 2022-12-27

**Authors:** Xiao Hu, Rita Yi Man Li, Kalpina Kumari, Samira Ben Belgacem, Qinghua Fu, Mohammed Arshad Khan, Abdulaziz A. Alkhuraydili

**Affiliations:** 1 School of Economics and Management, Wuhan University, Wuhan 430072, China; 2 Sustainable Real Estate Research Center, Department of Economics and Finance, Hong Kong Shue Yan University, Hong Kong 999077, China; 3 Faculty of Institute of Business and Health Management, Jinnah Sindh Medical University, Karachi 75500, Pakistan; 4 Business Administration Department, Business and Administration College, Princess Nourah Bint Abdulrahman University, Riyadh 11671, Saudi Arabia; 5 Department of Business Administration, Moutai Institute, Renhuai 564507, China; 6 Department of Accountancy, College of Administrative and Financial Sciences, Saudi Electronic University, Riyadh 11673, Saudi Arabia; 7 Department of Management, College of Administrative and Financial Sciences, Saudi Electronic University, Riyadh 11673, Saudi Arabia

**Keywords:** green leadership, leaders’ emotional intelligence, employees’ green organizational citizenship behavior, manufacturing and service industries of Pakistan

## Abstract

The green leadership (GL) concept has significantly gained popularity over the last decade. Consequently, more research has been conducted on this emerging leadership concept, emphasizing leadership styles that promote the green environment so that sustainable goals can be achieved. In the present research, leaders’ emotional intelligence (EI) is positioned as a mediating variable between GL and employees’ green organizational citizenship behavior (GOCB). The data of this research comprised managerial and non-managerial staff from the manufacturing and service industries. A PLS-SEM was used to evaluate the relationship between the various factors among 422 employees. The empirical findings indicated that GL and GOCB had a favorable and robust relationship. The results of the study also suggested that a leader’s EI mediates the influence of green leadership on their employees’ green organizational citizenship behavior. Green leadership is essential in creating sustainable environmental behaviors among employees. It can strengthen leaders’ EI, which successively helps them to garner positivity and foster an environment of mutual harmony and cooperation in the workplace to support pro-environmental policies. Overall, our study contributes to and advances previous studies and shows that green leadership plays a critical role in influencing a leader’s own EI which, in turn, predicts the green OCB of their employees in the workplace.

## 1. Introduction

Environmental effects have been a major concern for politicians, organizations, and scholars during the past few decades [1]. Laws and recent environmental pressures and efforts by NGOs have increased environmental expertise and sustainability awareness [2]. Green practices have been implemented into several organizations [3], including green leadership, products, and processes [4]. In this study, we are interested in how green leadership (GL) affects employees’ organizational behaviors and practices.

Transformational leadership theory is the most pertinent paradigm for comprehending how leaders might encourage green employee behaviors and improve an organization’s environmental performance [5,6]. This is due to transformational leaders’ focus on altering employee and organizational behaviors, including effective environmental management techniques [7]. GL is characterized as transformational management that strongly emphasizes motivating people to support environmental and green activities [8]. Several academics acknowledge that GL supports pro-environmental behaviors and improves environmental performance [9]. GL has emerged to combine HRM operations with environmental management. GL was found to be essential for fostering a sustainability culture in organizations [10]. To comprehend the interaction and relationship between leaders and their subordinates, we also draw on the leader–member exchange (LMX) theory and transformational leadership theory [11]. Several research investigations have studied the relationship between GL, green behaviors, and environmental performance. The main findings indicated that GL is a significant predictor of employees’ green behavior and environmental performance [12].

Recent research has revealed a strong link between GL, staff green habits, and environmental performance. However, studies on how the components above interact with Pakistan’s manufacturing and service sectors are scarce or nonexistent, according to scholars [13]. This study intends to close a knowledge gap in regards to the value of GL in encouraging green employee behaviors and green performance in Pakistani manufacturing and service-related enterprises.

In short, the current study intends to investigate the direct impact of GL on the overall organizational citizenship behaviors of workers towards the environment as well as the indirect impact of GL through leaders’ emotional intelligence in the manufacturing and service industries. This study has two key objectives: First, the study investigates the direct influence of GL on employees’ green organization citizenship behaviors. Second, it examines the mediating role of a leader’s emotional intelligence in the link between GL and the employees’ green organization citizenship behaviors in manufacturing and service-related businesses. To achieve an optimum environmental performance through GL and leaders’ emotional intelligence, the researchers utilized a theoretical framework and offer a set of pertinent implications for academics and practitioners in Pakistan’s manufacturing and service-related businesses. Thus, with this aim in mind, we came up with the following three research questions (RQs):

RQ1: How does leadership influence employees’ green organization citizenship behaviors in manufacturing and service-related organizations in Pakistan? 

RQ2: How does leaders’ emotional intelligence influence employees’ green organization citizenship behaviors in manufacturing and service-related organizations in Pakistan? 

RQ3: How does leaders’ emotional intelligence mediate between green leadership and employees’ green organization citizenship behaviors in manufacturing and service-related organizations in Pakistan? 

To accomplish the research goals and respond to the research questions, we organized our article as follows. By establishing the research constructs and going over how the research variables relate to one another, we give the theoretical framework for the study in Section 2. We describe the research methodology in Section 3, including the procedures we used to gather and examine the research data. We describe the research’s conclusions in Section 4. Section 5 outlines the conclusion along with management and theoretical implications of the study, identifies its limitations, and suggests areas for future research.

## 2. Literature Review and Research Hypothesis

This study builds its arguments on the social learning theory. It runs counter to B.F. Skinner’s work in a school of psychology known as behaviorism. It states that social behavior is learned through observing and modelling the behaviors of others [14]. Bandura proposed that people can pick up new habits just by watching others, an idea that runs counter to the focus of behavioral psychology, in which context and reinforcement shape behavior. According to the social learning theory, followers look up to leaders as role models for activities that will bring about both professional and personal success, and these leaders’ actions affect their subordinates. People learn positive traits, such as loyalty and teamwork, by observing and engaging with positive role models, as stated by Bandua and Walter [15].

Green leadership can inspire followers to practice civic virtues while carrying out their tasks and to engage in green conduct to advance the cause of sustainable development [16]. Green leaders promote observational learning about green beliefs and attitudes by modelling pro-environmental behavior and communicating sustainability standards and ideals to their followers. Green leaders set an example by acting pro-socially and responsibly toward the environment. Having green role models promotes green employee behaviors. Therefore, this paper uses the social learning theory to examine the relationship between green leadership and employees’ green OCB via leaders’ EI [11].

### 2.1. Green Leadership

According to the integration model of organizational or institutional behaviors, a leader and their team members can be evaluated based on their accomplishments (job outcomes), specifically their job performance or behaviors that contribute positively or negatively to the organization (organizational performance) [17]. Environmental leadership is an individual’s capacity to persuade and inspire other organizational members to engage in pro-environmental activities. Meanwhile, Gultom [18] defined green leadership more specifically as the ability of an individual’s charismatic leadership to transform and persuade others to engage in pro-environment actions. According to Arici and Uysal [19], making decisions with an eye on the environment is also a component of green leadership. Based on this knowledge, we may define “green leadership” as the capacity of a leader to choose environmentally friendly policies and persuade organizations to embrace such policies. Green leadership can impact interpersonal and organizational connections in an endeavor to accomplish sustainable environmental goals [8].

### 2.2. Employees’ Green Organizational Citizenship Behavior (GOCB)

GOCB is a discretionary behavior that improves environmental performance but is not officially rewarded by an organization [20]. Researchers have concentrated on organizational citizenship behaviors toward the environment in response to employees’ roles in the organization’s long-term development. GOCB occurs when staff members offer strategies to use fewer resources and energy or urge coworkers to carry out tasks in more environmentally friendly ways [21]. Furthermore, the term “GOCB” refers to a unique and adaptable social behavior that supports social well-being for value generation. “GOCB” in this study, however, refers to one person engaging in voluntary environmental activity at their discretion within the business but is not explicitly acknowledged and rewarded by the formal management structure.

### 2.3. Leaders’ Emotional Intelligence

Emotional labor is a word used by psychologists and social workers to describe the effort needed to control the way we express our emotions [22]. An awareness and understanding of one’s emotions, the capacity to control one’s emotions, and the capacity to express feelings in ways suitable for the situation are all recognized as essential to effective leadership, according to Case, Schwartz, and Ehasz [23]. Today’s leaders need emotional intelligence to improve their followers’ performance in general [24]. This type of constant leadership necessitates a leader with emotional intelligence who is aware of their own emotions, identifies them, comprehends them, and handles them to deal with each circumstance quickly and sincerely [25]. In the past, the business administration literature did not mention leaders’ emotions. Today, we understand how crucial it is for leaders to manage their emotions. We know that the capacity to manage one’s emotions, care for oneself and others, and foster positive relationships all contribute to being able to embrace necessary change [26]. Additionally, it better equips leaders to make the right decisions for themselves, other people, and their organizations, as well as to manage the demands of the workplace [27].

### 2.4. Green Leadership and Employees’ Green Organizational Citizenship Behavior

Major organizational changes start with leaders [28] who can set an example for their staff. Various groups are increasingly paying close attention to environmental concerns because of changes in the natural environment [21]. The influence of green leadership (GL) can help a firm’s employees adjust to a working system that cares about the environment [29]. In order to have an ecologically conscious firm that values nature, leaders need to be able to inspire employees’ motivation and boost employees’ green OCB. A green leadership style that prioritizes the environment is suitable for achieving it [30]. The participation of managers and employees is essential to the success of environmental conservation activities. Green leaders impact their followers’ creative thinking, pro-environmental behavior, and future outlook. By focusing on long-term goals, a green leader inspires followers to remain upbeat and transcend their present impressions [31].

Researchers have discovered a strong relationship between followers’ behaviors, their engagement in sustainable development, and green leadership styles [32]. For instance, Khan et al. [33] discovered that through mediating elements in the education sector, green leadership has a considerable impact on employee civic virtue. This kind of leadership encourages their followers to go above what is expected and frequently beyond what they think is feasible to promote sustainable development [34]. The organization embraces environmentally friendly practices with the support of leaders aware of environmental issues [35]. When green leaders serve as role models in sustainable activities, employees are motivated to behave and engage in sustainable activities. Employees are motivated to behave and act in environmentally responsible ways that benefit future generations and society. Employee commitment is significant because it supports the company’s primary goals by creating a suitable social and psychological climate in the workplace [36]. As a result, the primary tenet of the present study is that green leadership may precede followers’ green dedication and green OCB. The theoretical underpinnings of the social learning theory support this proposition. According to Bandura and Walters, 1977 social processes, such as modelling and observation, significantly impact how followers perceive and act in their environment. This study puts forward the following hypothesis in light of the above literature:

**H1:**  *Green leadership significantly influences employees’ green organizational citizenship behaviors.*

### 2.5. Green Leadership and Leaders’ Emotional Intelligence

Naturally, for a firm to succeed, it must cultivate talent that can enhance employee performance and further organizational performance, such as implementing changes [37]. Like other successful people, influential leaders have high levels of emotional intelligence, including self-awareness, self-control, motivation, empathy, and social competence. Leadership is now widely acknowledged to include the development of emotional intelligence. Emotional intelligence in corporate executives is thus one of the qualities that begins to grow [38].

However, these studies do not research the impact of green leadership on leaders’ emotional intelligence. According to earlier studies, people who take sustainable actions are more likely to experience positive than negative emotions. It is claimed that engaging in sustainable behavior makes people feel good about themselves, indicating that they perceive it to be intrinsically satisfying [39]. As a result, it is not surprising that there has been an increase in the literature that establishes a link between green leadership and an individual’s emotional intelligence [40]. Thus, the following hypothesis is proposed:

**H2:**  *Green leadership significantly influences leaders’ emotional intelligence.*

### 2.6. Leaders’ Emotional Intelligence and Employees’ Green Organizational Citizenship Behavior 

It is impossible to overstate how essential it is for institutions and organizations to adapt to survive and remain relevant in the age of globalization. As a result, it is essential for leaders in the 21st century to successfully lead change in their enterprises [41]. As no one likes giving up the security that comes with the status quo or giving up something they cherish, the transformation process is emotionally demanding [8] due to the difficulties involved in changing and the impacts of changes. To manage the process of change, there is a growing emphasis on emotional intelligence in leadership [42].

Organizations must develop and implement environmentally friendly procedures when faced with the issue of severe environmental pollution. Organizational green leaders and their EI have long been considered key players in corporate green management as practitioners of environmental management methods with the cooperation of their staff in the workplace [43]. Growing empirical research over the past ten years has emphasized the value of emotionally intelligent green leadership as a management strategy that enables commercial organizations to create and retain a competitive edge in achieving environmentally sound goals. Thus, emotionally competent green leaders positively influence followers’ thinking, pro-environmental behaviors, and outlook on the future [44]. Additionally, by concentrating on the long-term goal of sustainable development, they encourage followers to be optimistic and look beyond their previous perceptions [8]. Based on the given arguments, the following hypothesis is established:

**H3:**  *Leaders’ emotional intelligence significantly influences employees’ green organizational behaviors.*

### 2.7. Mediating Role of the Leaders’ Emotional Intelligence

**Figure 1:**
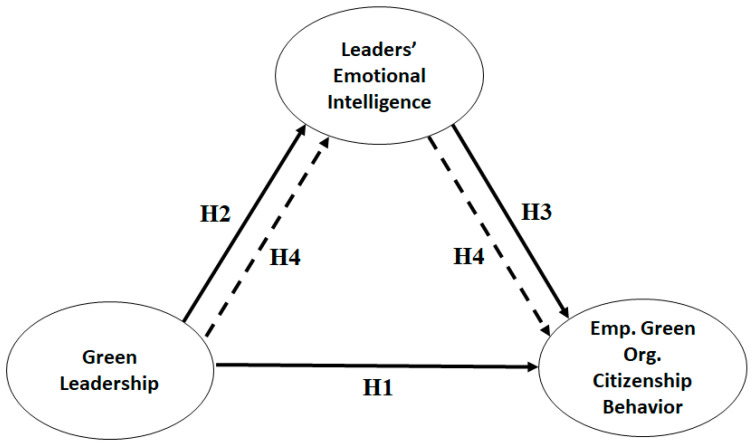
Theoretical Framework.

The impact of GL on employees’ organizational citizenship behaviors in favor of the environment has been supported by numerous studies [45]. The validity of GL as a predictor of green behavior among employees and environmental performance has also been confirmed. Recent research has investigated several mediators that may help explain how GL and green organizational citizenship practices are related. One study on restaurants discovered that CSR entirely mediates the connection between GL and environmental performance [46]. Another study on the industrial sector found that green innovation and green human resources management mediate the relationship between GL and environmental performance [47]. In addition, Nurwahdah and Muafi [25] demonstrated that leaders’ emotional intelligence had a mediating influence between employees’ corporate citizenship behaviors and green transformational leadership in a favorable and significant way. This study examines this relationship, and it is anticipated that leaders’ emotional intelligence positively mediates the relationship between GL and employees’ organizational citizenship behaviors related to the environment. This means that emotionally intelligent green leadership will be better able to promote an environmentally friendly culture within their organization. Hence, we hypothesize the following (Figure 1 contains the theoretical framework of the study):

**H4:**  *Leaders’ emotional intelligence significantly mediates the relationship between green leadership and employees’ green organizational citizenship behaviors.*

## 3. Research Method

### 3.1. Research Approach and Strategy

The study is based on a positivist (deductive) and logical approach. This study also used a survey technique related to the deductive approach and a common research method in business studies and management. Due to its ability to collect an enormous amount of data from a wide range of people in a relatively affordable manner, the method has grown in popularity [37].

### 3.2. Target Population and Sampling

The target population comprises all individuals working in service and manufacturing sectors within the cities of Karachi, Lahore, and Islamabad in Pakistan. Additionally, because the researchers concentrated on these three cities, they represent our target audience as accurately as possible because they represent various people from various cultural backgrounds. According to [48], a sample size of 30 to 500 respondents is adequate for most social science investigations with unknown population sizes. As a result, the authors gave 480 questionnaires to employees in Pakistan’s service and manufacturing industries. Appendix A presents the operationalized questionnaire.

The authors opted to use a convenience-based sampling technique to obtain the data because they could not obtain precise information regarding the total number of individuals employed in Pakistan’s service and industrial industries. When we do not have access to the entire population, the suggested sampling technique is regarded as the best course of action [49].

Additionally, employees with a minimum of one year of work experience were included in the data collection to ensure that they had the necessary time in the company to respond to the questions about their supervisor’s green leadership style, EI, and GOCB at work. The demographic factors in the study were the industry; the respondents’ gender, age, experience, and position in the organization; and organizational status. For a complete breakdown of the respondents’ demographics, see Table 1.

### 3.3. Data Collection Method

The current research followed an empirical approach to collect the data. Data were collected through a structured questionnaire using a Web-based platform, i.e., Google Forms, during the COVID-19 pandemic from the employees working in service and manufacturing industries located within Karachi, Lahore, and Islamabad in Pakistan.

Permission was requested from the organizations’ human resources departments to conduct the study within their workplace premises. The authors also asked for respondents’ business emails so they could contact them directly, if necessary, at a later stage of the investigation. To maintain objectivity in responses, the questionnaire included a cover letter explaining the study’s goal and assuring respondents that their responses would remain anonymous and that their participation in this study was entirely voluntary. A total of 480 individuals were sent standardized self-administered questionnaires through email (due to COVID-19, it was difficult to approach employees individually, and outsiders were also not allowed to enter the company). Only 245 completed questionnaires were initially returned after three weeks. A total of 210 more responses were obtained after gentle email reminders to receive the remaining respondents’ data. A total of 422 useful surveys were collected after eliminating questionnaires with missing or invalid responses. If the response rate was moderate, respondents would interpret the study’s relevance and rigor indirectly based on the supplied response rate. If respondents believed the study was significant and warranted their help, they would be more likely to return a questionnaire. Finally, PLS-SEM utilizing partial least squares software was used to examine 422 completed replies (Smart PLS version 3). Additionally, participants were asked to provide input on various supplied statements regarding their supervisor’s green leadership style, EI, and their GOCB by selecting the number that, on the provided scale, came the closest to reflecting their opinion.

### 3.4. Measurement of Variables 

When constructing the scales for variables, previously used and approved surveys were checked. Presentations were also delivered in English since it is the country’s official language of business. Thirty-three items were included in the survey scales used in this study to measure the three constructs. Five-point Likert scale with scores ranging from one (strongly disagree) to five (strongly agree) was used to examine the results entirely.

To measure green leadership, six items were used. The scale was initially created to assess servant leadership, but we have modified it and made a few amendments to make it more accurate from the standpoint of green leadership. Examples of sample items include “My manager can tell if something relating to the natural environment is going wrong” and “My manager makes environmental development activities a priority.” If a Cronbach’s alpha score of 0.70 and above indicates an internally consistent result [50], this study’s result was 0.80.

Emotional Intelligence (EI) was measured using 13 items developed by Wong et al. [51], in which an employee was asked to evaluate the current state of his leaders’ EI. The EI variable includes one’s emotional appraisal of self, emotional appraisal of others, use of emotion, and emotional regulation. Four items were used for each subscale. According to the study, the overall Cronbach’s alpha was 0.924. Examples of questions include *“My leader has a good sense of why he has a certain feeling most of the time” and “My leader always knows his friends’ emotions by their behavior.”*

GOCB was measured with a 10-item scale. The scale was first developed to evaluate employees’ OCB, but we have changed it and made a few adjustments to make it more precise regarding green OCB. Examples of sample questions include “I am happy to guide newcomers toward environmental issues and preservation” and “I am always ready to lend a helping hand to protect our climate and natural resources.” The overall reliability of the 10-item scale was 0.843 in this study.

### 3.5. Reliability and Validity 

Before releasing the link to the respondents, academic experts in human resource management, particularly those with expertise in the green leadership domain and pro-environmental behaviors, assessed the initial questionnaire to evaluate its content and face validity. They carefully examined the questionnaire’s content and its likelihood of measuring the study’s variables, including green leadership (GL), leaders’ emotional intelligence (EI), and employees’ corporate citizenship behavior (GOCB). 

According to Hinkin [52] advice, the authors first conducted pilot research to assess the questionnaire’s practicality, clarity, and appropriateness before conducting the full survey. Pre-tests were created to ensure that the measures were accurate, comprehensive, and valid. Cronbach’s Alpha (α) was used to assess the internal consistency of the research and the research instrument used in the pilot study. The results of the pilot study with 45 respondents showed that all the constructs had Cronbach’s alpha values higher than the allowable range of 0.7 [50]. These results demonstrated the consistency and dependability of the scales employed in this study. Additionally, the pilot study’s findings indicated that the suggested questionnaires are simple to grasp and can be completed in 7–8 min.

### 3.6. Common Method Bias

When all manifest variables are loaded into one factor in EFA without rotation using Harman’s one-factor approach, the variance for that single factor is 29.3% [53]. Meanwhile, three latent variables in the suggested model are accounted for, and the first factor covers 18.5% of the total variance. The percentage of variance for one factor is always less than 50%, indicating no serious common method bias. 

### 3.7. Analytical Approach

The data were analyzed with PLS-SEM. PLS-SEM has gained widespread esteem across various industries, including human resource management, strategic management, accounting, operations management, management information systems, marketing, supply chain management, hospitality, and tourism [54]. PLS-SEM is helpful in analyzing complicated latent variable models, according to Hair et al. [55]. As claimed by Hair et al., examining intricate higher-order models offers high predictive potential [56]. When evaluating the structural model, PLS-SEM has the advantage of studying latent constructs through path analysis and emphasizing the explanation of variance in dependent variables. PLS-SEM uses two different models to examine data. The measurement model comes first and details the connection between latent and observable variables. The second is a structural model that looks at the relationships between the latent variables [57]. A similar approach has been applied in researching the factors that affect people’s willingness to share construction safety knowledge via Web 2.0, IoT, and mobile apps.

## 4. Results and Discussion

Using a two-stage measurement and structural model, the gathered data were examined. This study evaluated the reliability of the constructs using Cronbach’s alpha (CA) coefficient. The measurement reliability of each construct was examined using composite reliability (CR).

### Result and Interpretation

Descriptive statistics, Cronbach’s alpha, and a composite reliability analysis were used to assess the associations among the latent variables for the measurement model, and the results showed that all the variables had a substantial positive link with one another (See Table 2). Cronbach’s alpha values larger than 0.7 are advised to be the threshold for acceptance, according to Petersom [58]. The overall dependability of all the metrics was more than 0.7 and considered acceptable.

The authors also checked the convergent and discriminant validities to ensure that all the constructs were noticeably distinct. According to Hu and Bentler [59], if the average variance extracted (AVE) value is more than 0.4, it should be taken as evidence of convergent validity. The AVE analysis identified all constructs with values larger than 0.5. Table 2 includes the results of this measurement. Additionally, if the inter-item correlation is low, it indicates that the items originate from different domains and that deletion is necessary to reduce errors and unreliability [58]. Items with loading lower than 0.40 should be deleted, according to [60]. All the Cronbach’s alpha coefficients were higher than the advised level of 0.6 after eliminating items SL1, EI2, EI3, EI8, OCB6, OCB9, and OCB10 [57].

The Fornell–Larcker criterion matrix is shown in Table 3. Each measurement has larger AVE squared roots than its correlation with the other latent variables, which means the acceptable discriminant validity threshold is also met [61]. Since the results for each construct’s row or column did not exceed the value in the diagonal, discriminant validity was achieved [57]. The authors then performed a Heteotriat–Monotrait (HTMT) test of the correlation ratio to cross-examine the validity. Henseler et al. [62] suggested that the maximum allowed value of HTMT should be 0.85. As per Table 4, the values fully complied with the suggested criteria, representing the appropriateness of the validity.

The structural model was assessed by evaluating the total variance or R2, *p*-value, t-statistic, and beta. The path analysis demonstrated that none of the control variables (age, gender, education, and position) significantly affected the OCB. Table 4 and Table 5 show the path coefficients (β) with their respective t-statistic, *p*-values, and R2 values. Table 4 and Table 5 show three paths that have significant positive relationships among the latent variables. The highest positive significant path relationship was between GL and GOCB (β = 0.275, *t* = 2.412, *p*-value 0.003). GL and EI also indicated a significant positive relationship (β = 0.232, *t* = 2.208, *p*-value 0.007). Similarly, EI and GOCB also presented a significant positive relationship (β = 0.233, *t* = 2.246, and a *p*-value of 0.008). However, the least positive significant path relationship was identified between EI as a mediator between SL and OCB (β = 0.029, *t* = 2.141, *p* < 0.05). Thus, H1, H2, and H3 are fully supported. However, H4 is partially supported.

**Figure 2:**
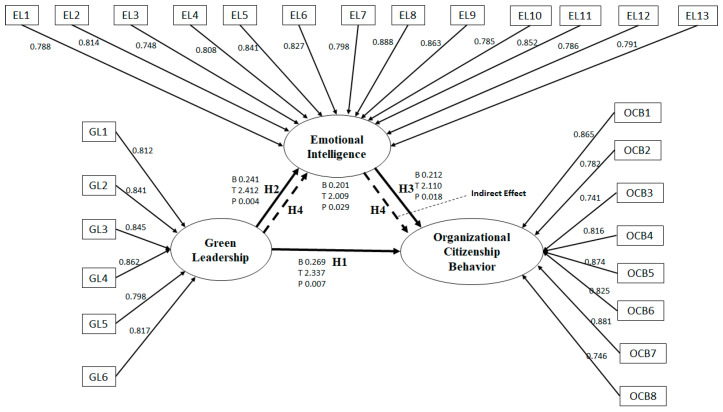
Results of PLS-SEM analysis.

With the use of the bootstrapping technique and a PLS-SEM analysis, this study used direct, indirect, and total effect measurements to examine the mediating role of leaders’ emotional intelligence (EI) between green leadership (GL) and employees’ green organizational citizenship behavior (GOCB) [63]. In addition, the PLS technique was used to determine the coefficient values. Figure 2 represents the structural model.

## 5. Conclusions, Discussions, Implications, and Future Research 

### 5.1. Conclusions and Discussions

Studies have linked GL to a willingness to go the extra mile, which indicates that it is an important factor in encouraging GOCB in employees in the workplace [64]. Additionally, this study aimed to investigate the link between GL and GOCB through the mediating role of leaders’ EI.

This study also concludes that green leadership positively and significantly affects employees’ green organizational citizenship behaviors with a *p*-value of 0.007. Significant organizational changes will start with leaders who can set an example for their staff. The influence of green leadership (GL) in a firm can serve as an illustration for workers to become acquainted with a functioning system that can still care about the environment [65]. The demand for leaders who can inspire employees arises from the requirement to boost employees’ green OCB in order to achieve an environmentally conscious firm that values nature. This can be accomplished with an environmentally conscious leadership approach. The green leadership philosophy inspires subordinates to be model leaders. Green leaders have a high level of organizational civic behavior and a favorable impact, as per Widisatria and Nawangsari [35], so that they can prioritize the needs of the business before their own. As a result, the company’s will be more focused on environmentally friendly OCBs. The development of green leadership will positively impact people who care about the environment and management’s ability to live sustainably [36]. Significant organizational improvements will start with leaders who can set an example for their staff. Due to the deteriorating state of the environment, businesses are making efforts to transform to be environmentally friendly and to urge their teams to adopt green behaviors. A degradation in environmental quality necessitates green leadership that is based on concern for environmental sustainability today.

The researchers of this study also deduced that, with a *p*-value of 0.004, green leadership has a favorable and significant impact on leaders’ emotional intelligence. According to this, leaders’ emotional intelligence increases in proportion to how concerned they are about the environment and decreases in proportion to how green their leadership is. The findings of this study are consistent with the theory advanced by Nurwahdah and Muafi [25], according to which a leader with a green leadership spirit invites everyone to make significant changes. As per Rajee, Umma, and Kengatharan [66], a strong emotional bond between a leader and his subordinates can help an organization or business advance. Batik producers and the chairman of the Kebon Indah Community have an emotional tie that is similar to a family bond, and this emotional comfort has helped these batik makers expand their market reach, since working together strengthens and promotes the development of each other’s brand. An organization’s senior management must always adopt this attitude of mutual trust and support with its staff.

Similarly, leaders’ emotional intelligence has been shown to influence employees’ organizational citizenship behaviors significantly and positively in Pakistan’s manufacturing and service sectors, with a *p*-value of 0.018. This might mean that the more emotionally intelligent leaders are, the more ecologically conscious employees will behave at work; conversely, the less emotionally intelligent leaders are, the less environmentally conscious employees will behave at work. Employees who can control their emotions and those of others will be able to adjust to a rapidly changing environment more swiftly [41]. Emotional intelligence can be defined as the capacity to recognize, comprehend, and constructively manage emotions in oneself and others to reduce stress and communicate successfully. Individuals with emotional intelligence are better able to sympathize with others and deal with difficulties. John Mayer is credited as the originator of the phrase ‘emotional intelligence.’ The results of this study indicate that emotionally intelligent leaders in the manufacturing and service sectors will be better able to implement their staff members’ green practices because they already have a robust emotional sense and a sense of ownership ingrained in their minds Nurwahdah and Muafi [25] assert that leaders’ ability to control their emotional intelligence will impact how other people view them. If an organization and its employees get along well, the employees will contribute more to the organization. For instance, they may adopt green corporate citizenship behaviors. A person’s love of nature and their understanding that all people who coexist with nature must not harm it will contribute to their EI and help to encourage green business practices [67]. Dimitrov [68] contends that a company’s OCB level will rise if people are motivated to perform tasks voluntarily.

Additionally, leaders’ emotional intelligence also showed promise as a significant mediating factor between green leadership and employees’ green corporate citizenship actions. Emotionally intelligent company leaders will impact several organizational elements, including their capacity to foster commitment, create positive working relationships with staff members, and raise employee happiness levels [69]. Seeing green leaders as role models and using emotional intelligence as a mediator have increased green OCB in organizations, according to Irshad and Hashmi (2014). The study’s findings revealed a significant link between green leadership (X1) and organizational green citizenship behaviors (Y) through emotional intelligence (Z). The association between green leadership and green organizational citizenship behaviors is therefore mediated by emotional intelligence as a variable. Strong green leaders act as mentors or advisers to aid employees, their needs, personal growth, and learning. Additionally, they can encourage one another to put their professional interests ahead of their personal ones by building mutual trust. Expertise and abilities in environmental management are greatly influenced by effective leadership; if a leader and their team get along well, they can set a positive example that others can follow [70]. The study’s findings corroborate those of [71], who discovered that emotional intelligence substantially impacts the relationship between transformational leadership and OCB and can serve as a mediator in that relationship [45,72]. The study’s findings concur with those of [70], who discovered that a transformational leadership style positively impacted willingness to engage in additional roles in the workplace, making it more meaningful.

### 5.2. Theoretical Contributions

In three ways, the findings of this study add to the body of knowledge on green leadership. First and foremost, it demonstrates the benefits of green leadership throughout Pakistan’s manufacturing and service industries. The relationship between green leadership and GOCB has been extensively studied empirically over the last decade, but few studies have been conducted in the Pakistani context. The results of this study demonstrate that green leadership has a significant impact on employees’ green OCB. It indicated that green leadership could increase employees’ involvement in voluntary, environmentally friendly activities, helping achieve the objective of protecting the environment on the level of the individual institution.

Second, to our knowledge, this research is the first to look at the relationship between green leadership and leaders’ emotional intelligence and has proven that green leaders have high levels of emotional intelligence (EI). Several empirical studies have also shown that green leadership improves employees’ mental and emotional well-being [25]

In addition to providing evidence on how green leaders cultivate GOCB amongst their employees, our study’s final contribution is the incorporation of leaders’ EI as a mediator variable. Leaders’ emotional intelligence (EI) was not previously studied as a mediator between the given constructs of the current study. Serving as a green leader may play a crucial function in enhancing leaders’ EI, which may increase employee GOCB and green organizational performance. According to the proposed model, which connects green leadership with employees’ GOCB, it has therefore been concluded that green leaders can help their employees to develop organizational citizenship behaviors for the environment (OCBE) that are voluntary and not explicitly recognized by a formal reward system, contributing to more effective environmental management by the organization [29]. Additionally, it has also been shown that leaders’ EI is a significant mediator between green leadership and employees’ GOCB.

### 5.3. Practical Contributions

According to our managerial results, organizations, particularly those in Pakistan’s manufacturing and service sectors, may not directly influence work performance, such as overall organizational performance and organizational green performance. Instead, they could build a green strategy and incorporate it into green HR practices, which would create an ideal atmosphere for the growth of micro-foundations, such as environmentally specific green leadership among supervisors and OCBEs among front-line workers.

According to our research, one micro-foundation for employee OCBEs is supervisors’ environmentally conscious leadership, which helps transfer green management activities (the fusion of green strategy and green HR practices) into OCBEs among their followers. Organizations in the service and manufacturing sectors should implement a variety of hiring, training, feedback, recognition, and information-sharing procedures to foster supervisors with green leadership styles as well as green values, green-related knowledge and skills, and green behaviors. Additionally, practices for selecting, training, and rewarding leaders should emphasize skills and qualities related to enhancing their emotional intelligence (EI), which may include, among other things, their conceptual abilities, empowerment of others, assistance, emotional recovery, and ethical actions [25]. Another important point to emphasize is that in order to foster environmentally specific green leadership, businesses must monitor the implementation of a number of green HRM practices as well as their alignment and interaction with the green strategy. Environmentally specific green leadership is becoming more common among managers. It not only communicates to followers the signals and cues from the green strategy and green HR practices, but it also complements and strengthens the effectiveness of green HR practices by setting an example of green values and fostering an environment that encourages employee OCBEs.

### 5.4. Limitations and Future Directions

Despite the researchers’ unrelenting and determined efforts to give sufficient data and evidence to support the study, it is nonetheless essential to draw attention to its flaws so that they can be avoided in future studies.

The generalizability of this study has some restrictions. First, the study’s generalizability was hampered by the fact that only employees of the service and manufacturing sectors in Karachi, Lahore, and Islamabad provided the data. Therefore, the findings might not apply to all other industries. Therefore, other sectors of the various businesses should also be considered in future research to explore the relationship between the supplied constructs in greater detail. These industries could include those in the social sector, health, and defense.

The second drawback of this study is the use of leaders’ EI as the sole mediator in attempting to create a relationship between the supplied variables. There is still an opportunity to include many more mediators to gain more profound knowledge of the relationship and association between green leadership and employees’ GOCB.

The study’s third drawback is that it is cross-sectional in nature. We need to undertake longitudinal studies in the future to conclusively show the cause-and-effect relationship between green leadership and employees’ GOCB because cross-sectional studies cannot establish strong cause-and-effect relationships.

Additionally, because employees were the only source of information used to examine the various variables in this study, any inefficiency or shortcoming in that source could skew the outcomes of the study. Therefore, it is advised that the data to be surveyed should be gathered from numerous sources in the future to reduce the effects of any such disparity. However, because all of the mentioned components were evaluated using subjective criteria, it may be challenging to provide accurate, reliable, and valid results. Therefore, to obtain more accurate and valid results, future researchers should seek to employ objective metrics.

Finally, the study is limited by the small number of respondents, even with an acceptable minimum respondent number (300). This limitation on time and money restricts our capacity to undertake an exhaustive analysis of all organizations. Even though the results are noteworthy and significant, the study’s statistical power and generalizability are likely to be lacking due to the small sample size. In future studies, researchers should appropriately address this problem by enlarging their sample sizes.

## Figures and Tables

**Table 1:** Demographic Characteristics of Respondents (*n* = 422).

Particulars	Description	Values	Percentage
Type of Industry	Manufacturing	173	41%
	Services	249	59%
Gender	Female	215	51%
	Male	207	49%
	Prefer not to say	4	1%
Age	Less than 20	135	32%
	20–30	203	48%
	31–40	59	14%
	41–50	8	2%
	50+	13	3%
Years of experience	Less than 5	131	31%
	5–10	207	49%
	11–15	68	16%
	16–20	6	1.50%
	More than 20	11	2.60%
Position within the organization	Operational staff	165	39%
	Junior management	152	36%
	Middle management	97	23%
	Top management	9	2.08%
Organizational status	Public	194	46%
	Private	228	54%

**Table 2:** Factor Loading, Mean, SD, CA, CR, AVE.

Variables	Items	Loading	Mean	SD	CA	CR	AVE
**Green Leadership**	GL 1	0.656	0.356	0.048	0.843	0.881	0.555
	GL 2	0.724					
	GL 3	0.760					
	GL 4	0.830					
	GL 5	0.752					
	GL 6	0.732					
**Emotional Intelligence**	EI 1	0.663	0.375	0.040	0.924	0.933	0.521
	EI 4	0.648					
	EI 5	0.730					
	EI 6	0.620					
	EI 7	0.805					
	EI 8	0.737					
	EI 10	0.765					
	EI 11	0.763					
	EI 12	0.694					
	EI 13	0.740					
	EI 14	0.739					
	EI 15	0.786					
	EI 16	0.663					
**Green Organization Citizenship Behavior**	GOCB 1	0.685	0.469	0.035	0.843	0.881	0.521
	GOCB 2	0.805					
	GOCB 3	0.817					
	GOCB 4	0.778					
	GOCB 5	0.648					
	GOCB 7	0.748					
	GOCB 8	0.826					

Notes: SD = Standard Deviation, AVE = Average Variance Extracted, CR = Composite Reliability. Items GL1, EI2, EI3, EI8, GOCB6, GOCB9 and GOCB10 were deleted due to low loadings.

**Table 3:** Fornell–Larcker Criterion (Discriminant validity).

Variables	EI	OCB	SL
**EI**	**0.719**		
**GOCB**	0.452	**0.732**	
**GL**	0.458	0.499	**0.751**

**Note(s):** EI, Emotional intelligence; GOCB, Green Organizational citizenship behavio; GL, Green Leadership.

**Table 4:** Heteotriat–Monotrait (HTMT) Ratio of Correlation.

HTMT						
LD						
SEA	0.524					
OEA	0.598	0.551				
UoA	0.499	0.476	0.54			
RoA	0.499	0.488	0.452	0.732		
OCB	0.61	0.529	0.458	0.499	0.541	
	LD	SEA	OEA	UoA	RoA	OCB

**Table 5:** PC, TS, PV, R^2^.

Variables	Β	T-Statistic	*p*-Value	Result
GL toward GOCB	0.269	2.337	0.007	Accepted
GL toward EI	0.241	2.412	0.004	Accepted
EI toward GOCB	0.212	2.110	0.018	Accepted
EI b/w GL and GOCB	0.201	2.009	0.029	Partially Accepted

**Note(s):** PA, path-coefficient; β, beta; TS, t-statistic; PV, *p*-value.

## Data Availability

The data will be made available on request from the corresponding author.

## Appendix A. Questionnaire

Please circle your desired response as to what extent you are agree with that statement: For example, if your response is 4 (Agree) then draw a circle around 4.

**Table:**

S. No	Items (Green Leadership)	Strongly Disagree	Disagree	Neutral	Agree	Strongly Agree
01	My manager can tell if something relating to the natural environment is wrong.	1	2	3	4	5
02	My manager makes environmental development activities a priority.	1	2	3	4	5
03	My manager talks to us about environmental issues and preservation.	1	2	3	4	5
04	My manager emphasizes the importance of protecting the natural climate and resources.	1	2	3	4	5
05	My manager considers organizational and environmental interests while making decisions.	1	2	3	4	5
06	My manager gives us freedom to practice new ideas that could potentially conserve resources.	1	2	3	4	5
07	My manager would not compromise environmental principles in order to achieve success.	1	2	3	4	5
**S. No**	**Items (Emotional Intelligence)**	**Strongly ** **Disagree**	**Disagree**	**Neutral**	**Agree**	**Strongly** **Agree**
**Self-emotion appraisal (SEA)**						
1	My leader has a good sense of why he has certain feelings most of the time.	1	2	3	4	5
2	My leader has a good understanding of his own emotions.	1	2	3	4	5
3	My leader really understands what he feels.	1	2	3	4	5
4	My leader always knows whether or not he is happy.	1	2	3	4	5
**Others’ emotion appraisal (OEA)**						
1	My leader always knows his friends’ emotions from their behavior.	1	2	3	4	5
2	My leader is a good observer of others’ emotions.	1	2	3	4	5
3	My leader is sensitive to the feelings and emotions of others.	1	2	3	4	5
4	My leader is good understanding of the emotions of people around him.	1	2	3	4	5
**Use of emotion (UOE)**						
1	My leader always set goals for himself and then try his best to achieve them.	1	2	3	4	5
2	My leader always tell himself that he is a competent person.	1	2	3	4	5
3	My leader is a self-motivated person.	1	2	3	4	5
4	My leader always encourages himself to try his best.	1	2	3	4	5
**Regulation of emotion (ROE)**						
1	My leader is able to control his temper and handle difficulties rationally.	1	2	3	4	5
2	My leader is quite capable of controlling his own emotions.	1	2	3	4	5
3	My leader always calms down quickly when he is very angry.	1	2	3	4	5
4	My leader has good control of his own emotions.	1	2	3	4	5
**S. No**	**Items (Organizational Citizenship Behavior)**	**Strongly** **Disagree**	**Disagree**	**Neutral**	**Agree**	**Strongly** **Agree**
1	I am happy to guide newcomers toward environmental issues and preservation.	1	2	3	4	5
2	I am always ready to lend a helping hand to protect our climate and natural resources.	1	2	3	4	5
3	I am always willing to help out in the effort to preserve the environment and its resources.	1	2	3	4	5
4	I am conscious of how my actions may affect resources.	1	2	3	4	5
5	In order to succeed, I keep myself informed about organizational environmental principles.	1	2	3	4	5
6	I attend meetings that are optional but that aid in dealing with environmental issues and preservation for my organization.	1	2	3	4	5
7	I can see things that are wrong with what my company is doing regarding the environment.	1	2	3	4	5
8	Normally, I give precedence to environmental development initiatives.	1	2	3	4	5
9	I abide by company policies that support my organization’s efforts to address environmental issues and preserve the environment.	1	2	3	4	5
10	I go above and beyond at work in order to preserve the environment and its resources.	1	2	3	4	5
